# Contribution of Experimental Animal Research Studies to the Emergency Medicine Literature

**DOI:** 10.1155/2019/8578674

**Published:** 2019-04-01

**Authors:** Umut Ocak, John H. Zhang

**Affiliations:** ^1^Department of Physiology and Pharmacology, Loma Linda University School of Medicine, Loma Linda, CA 92350, USA; ^2^Department of Anesthesiology, Loma Linda University School of Medicine, Loma Linda, CA 92350, USA; ^3^Department of Neurology, Loma Linda University School of Medicine, Loma Linda, CA 92350, USA; ^4^Department of Neurosurgery, Loma Linda University School of Medicine, Loma Linda, CA 92350, USA

## Abstract

The aim of this study is to provide a detailed analysis of emergency medicine (EM) research literature to unveil the trends while underlining the importance of experimental research for all territories of science. To this end, the experimental animal research articles published in EM journals indexed to the Science Citation Index Expanded database with a date of publication between January 1, 2008, and December 31, 2017, were reviewed retrospectively. All data regarding the journal name, publication year, country, department and institution of the first author, subject species, type of the experimental model, target organ/system/functions, evaluation method, outcome measures, and citation counts were noted. Resultantly, a total of 736 articles were found to be published in 18 journals. Resuscitation (n=285, 38.7%) had the highest number of articles followed by Injury (n=143, 19.4%), Turkish Journal of Trauma and Emergency Surgery (n=128, 17.4%), and American Journal of Emergency Medicine (n=63, 8.6%). The USA was the largest contributor with 199 studies (27%). The department of the first author was EM in 190 (28.8%) of the reports. Various versions of cardiac arrest models were applied in 257 (34.9%) studies while brain (n=101, 13.7%) was the most commonly explored area. The main outcome measures were clinical outcomes/survival rates (n=408, 55.43%). The molecular mechanisms of the injury were evaluated in 37 (5%) of the studies. In conclusion, experimental animal studies are essential in the progress of contemporary scientific knowledge. EM journals should encourage and consider giving more place to experimental research given their undisputed worth and potential future contributions to science, including the field of EM.

## 1. Introduction

Bibliometric studies provide collective and summarized data regarding the scientific productions of a particular country, journal, or author as well as the articles published in a specific field or topic. Recently, there is growing interest in quantitatively analyzing written publications by web-based bibliometric studies, presumably due to the rapidly expanding medical literature.

Emergency Medicine (EM) has the ability to cover all medical fields and also has an assembler role among subspecialties. Accordingly, being a considerable contributor to contemporary medical literature renders EM one of the most exciting areas for bibliometric analysis. To date, featured topics of EM literature have been defined by focusing on the most influential articles or highly cited journals [1–4]. However; directing the most cited works per se may result in further overlooking of less studied or less attended issues given the substantial body of knowledge of EM, encompassing numerous research topics. In that regard, since the headlines of leading interests have already been approximated, more detailed analysis on particular subfields in order to orient future studies are mandatory.

For all territories of science, experimental studies are the gates opening to solutions while encouraging advanced inquiries. Moreover, experimental animal research helps obtain information which is not possible to acquire in other ways while allowing the application of adapted models of certain pathologies that would not be plausible to perform on humans. Consequently, for more than a decade, animal researches have been of great importance in all aspects of science given their substantial contributions to the understanding of disease processes along with the development of drug therapies [5], vaccines [6], and surgical techniques [7].

Currently, the contribution of experimental animal studies to the EM literature and the trends of these studies are unknown. For this end, we aimed to provide a detailed analysis of EM journals to help unveil the propensities, distribution and shortcoming topics regarding experimental research which would also reflect the foremost challenges of daily practice. Resultantly, collaboration among researchers could be supported and facilitated while forcing them to ask new scientific questions with the strength of the evidence associated with their area of interest.

## 2. Methods

### 2.1. Study Design

This study was conducted in July 2018 as a retrospective, descriptive study of the experimental animal research papers published in EM journals indexed to the Science Citation Index Expanded (SCI-E) database. Institutional review board approval was not needed for this study as the data evaluated was publicly available and no human or animal subjects were involved.

The subject categories of SCI-E of Journal Citation Reports 2017 provided by the Institute for Scientific Information revealed a total of 27 journals respecting the field of EM. The journals published in a language other than English were excluded. Then, every article in each issue of these journals with a date of publication between January 1, 2008, and December 31, 2017, was reviewed manually through the official journal websites. Original articles reporting experimental animal studies were included in the study while human studies that did not involve an animal subject were excluded.

All data were analyzed based on the journal name, publication year, article title, country, department and institution of the first author, subject species, type of the experimental model, target organ/system/functions, type of the evaluation method, outcome measures, and citation counts. Institutions were further categorized as ‘*education centers*' when the institution of the first author was linked to a university, medical college, or training hospital; whereas it was considered as ‘*other medical/scientific organizations'* when the institution was a government or state hospital, medical institute, surgery clinic, research institute, science center, etc. not linked to a university or medical college. Similarly, evaluation methods were categorized as outcome/survival, histopathological, blood/serum, radiologic, polymerase chain reaction, and electrophysiologic studies. Variables constituting less than 1% were considered as ‘others' in all categories.

The impact factors of the journals were obtained from 2017 Journal Citation Reports and Thomson Reuters Web of Science Core Collection (Philadelphia, Pennsylvania, USA) was used to identify the citation status of the articles [8].

### 2.2. Statistics

Since our aim was to describe the trends and distribution of the studies along with their contents and not to perform a comparative investigation in order to test a hypothesis, we did not proceed with statistical analysis. Thus, the results were simply presented as number of quantifications and percentages.

## 3. Results

### 3.1. Characteristics of the Articles

Excluding those with a publication language other than English revealed 24 SCI-E EM journals for further assessment. Reviewing all issues published between 2008 and 2017 revealed a total of 736 articles matching our inclusion and exclusion criterion in 18 of the journals.* Academic Emergency Medicine, American Journal of Emergency Medicine, Annals of Emergency Medicine, Burns & Trauma, Emergencias, BMC Emergency Medicine, European Journal of Emergency Medicine, European Journal of Trauma and Emergency Surgery, Hong Kong Journal of Emergency Medicine, Injury, Journal of Emergency Medicine, Prehospital and Disaster Medicine, Prehospital Emergency Care, Resuscitation, Scandinavian Journal of Trauma, Resuscitation & Emergency Medicine, Signa Vitae, Turkish Journal of Trauma & Emergency Surgery, *and* World Journal of Emergency Surgery* were the journals which were found to publish at least 1 experimental animal study during this period.

**Figure 1:**
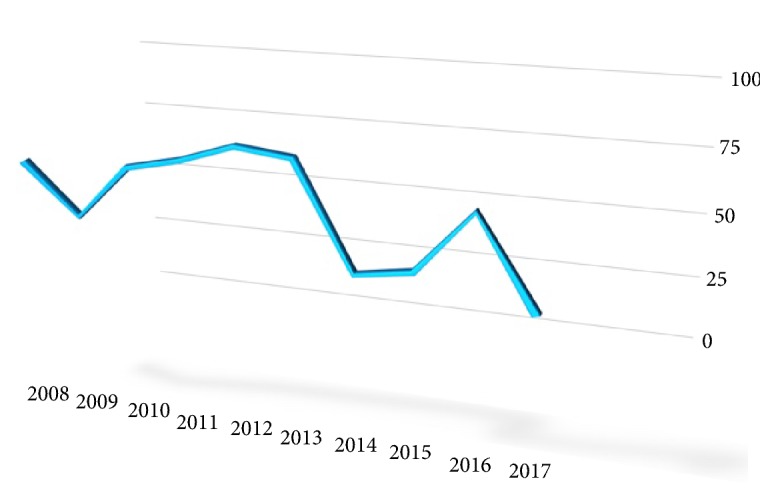
Distribution of the experimental animal research studies published in SCI-E emergency medicine journals according to years.

More than half of the reports were published in the first half of the 10-year period, between 2008 and 2012 (n=405, 55%), compared to the second half, between 2013 and 2017 (n=331, 45%). Consistently, although there was generally a gradual increase in the number of studies during the first 5 years, there was a sharp decrease in 2014 with 55 articles, which remained low since then (Figure 1).

The journal with the highest number of published articles was Resuscitation, with 285 (38.7%) followed by Injury, with 143 (19.4%) and the Turkish Journal of Trauma and Emergency Surgery, with 128 (17.4%). The American Journal of Emergency Medicine and Academic Emergency Medicine contributed with 63 (8.6%) and 37 (5%) studies, respectively (Table 1).

A total of 39 countries contributed to the publications regarding experimental animal studies. The USA was the largest contributor with 199 articles (27%), followed by Turkey (n=147, 20%) and China (n=102, 13.9%) (Table 2).

### 3.2. Characteristics of the Experimental Studies

Five-hundred and eighty (72.2%) of the studies were conducted at education centers while 156 (20.8%) were conducted in other medical/scientific organizations. In 75 of the studies (10.2%), the department of the first author was not specified. Among the articles in which the department of the first author was available (n=661), EM had the major contribution with 190 articles (28.8%) followed by anesthesiology (n=107, 16.2%). Seventy-two (10.9%) and 64 (9.7%) of the articles were published by the departments of orthopedics and surgery, respectively.

Rodents were the primary study population in 367 of the studies (49.9%) followed by swine (n=272, 37%). Various versions of cardiac arrest models were applied in 257 (34.9%) of the studies whereas models of inflammation, infection, ischemia or trauma of the major organs such as lungs, kidneys, liver, etc. were performed in 87 (11.8%). Hemorrhage and bone fracture models were performed in 81 (11%) and 66 (9%) of the studies, respectively. Brain (n=101, 13.7%) was the most commonly explored area in the studies which targeted a specific organ or system and examined the consequences of the injury, followed by hemodynamic parameters such as mean arterial blood pressure, heart rate, and blood flow (n=94, 12.8%). Cardiac functions and bones were targeted in 80 (10.9%) and 61 (8.3%) of the studies, respectively. The main outcome measures were clinical outcomes and survival rates in 408 (55.43%) of the studies. Three-hundred and fifty-four (48.1%) of the experiments included histopathological evaluation while 124 (16.8%) reported their results of blood/serum analysis. The majority of the studies were in vivo (n=717, 97.4%) while 7 (1%) were in vitro and 12 (1.6%) were both. The results of interventional experiments examining the molecular mechanisms of the injury in order to explore the affected signaling pathways were available in 37 (5%) of the studies. Characteristics of the experimental studies reported in the articles are summarized in Table 3.

### 3.3. Citation Counts

**Figure 2:**
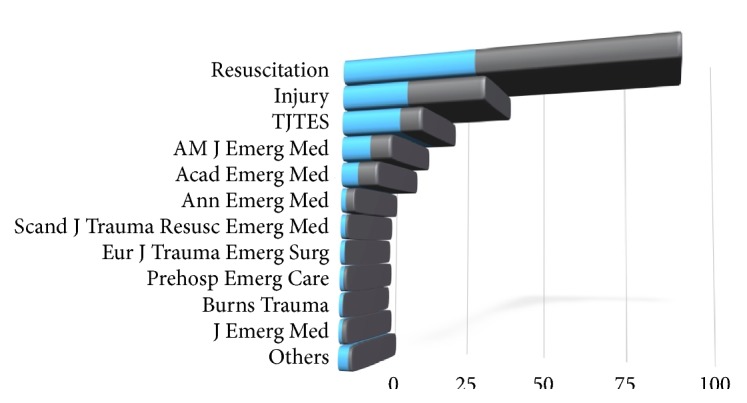
Distribution of the articles and their citation counts according to journals (blue bars: percentage of the articles per journal, grey bars: percentage of the citations per journal).

The experimental animal research articles in EM journals received a total of 7207 citations. The number of citations ranged between 0 and 86. Seventy-two (9.8%) of the articles were not cited at all. Resuscitation gained the most citations (n=3787, 52.5%) followed by Injury (n=1588, 22%) and Turkish Journal of Trauma and Emergency Surgery (n=455, 6.3%). The American Journal of Emergency Medicine received 451 citations (6.2%) while Academic Emergency Medicine gained 426 (5.9%). The distribution of the articles and their citation status per journal are presented in Table 1 and Figure 2.

Acad Emerg Med: Academic Emergency Medicine, Am J Emerg Med: American Journal of Emergency Medicine, Ann Emerg Med: Annals of Emergency Medicine, Burns Trauma: Burns & Trauma, Eur J Trauma Emerg Surg: European Journal of Trauma and Emergency Surgery, J Emerg Med: Journal of Emergency Medicine, Prehosp Emerg Care: Prehospital Emergency Care, Scand J Trauma Resusc Emerg Med: Scandinavian Journal of Trauma, Resuscitation & Emergency Medicine, TJTES: Turkish Journal of Trauma and Emergency Surgery

Among the countries that contributed, the USA gained the most citations (n=2663, 37%) followed by China (n=1102, 15.3%) and Turkey (n=757, 10.5%). Worldwide productivity based on the total number of articles and citation counts is presented in Table 2.

The citation rate for the top 25 cited articles ranged from 37 for Bebarta et al.* (Hydroxocobalamin and Sodium Thiosulfate Versus Sodium Nitrite and Sodium Thiosulfate in the Treatment of Acute Cyanide Toxicity in a Swine (Sus scrofa) Model)* [9] to 86 for Jia et al.* (Improving Neurological Outcomes Post-Cardiac Arrest in a Rat Model: Immediate Hypothermia and Quantitative EEG Monitoring)* [10]. The USA had the most articles in the top 25 cited list with 13 while Norway and China each had 2 and Malaysia, Austria, Taiwan, Sweden, Switzerland, Germany, Netherlands, and Finland each had one. Ten (40%) of these studies conducted cardiac arrest models (Table 4).

Acad Emerg Med: Academic Emergency Medicine, Am J Emerg Med: American Journal of Emergency Medicine, Ann Emerg Med: Annals of Emergency Medicine

## 4. Discussion

Bibliometrics, the quantitative analysis of a particular field or topic, provides valuable information about the trends and distribution of specific issues over a certain time period. Moreover, by citation analysis, the most valued studies with a potential to influence the world of science can be underlined since the number of citations is currently accepted as the most important indicator of the impact of a scientific paper [11].

Until today, bibliometric analyses based on the citation status of articles related to a certain field of medicine have been performed widely, including EM [3, 4, 12–16]. Therefore, the most interesting topics of general EM literature were identified before, revealing toxicology, traumatology, cardiovascular and resuscitation medicine as the most influential. Indeed, the focus was mainly in the original articles and clinical reports rather than reviews and experimental studies [3, 4].

The additives of animal experiments to scientific knowledge and human well-fare are beyond dispute. In addition to these contributions from past to present, experimental research also has the ability to critically guide future studies. Hence, the present study was accomplished to analyze this crucial branch of science in the EM territory. To this end, evaluation of the relevant journals led us to identify a number of topics. For example, the most commonly studied themes were cardiac arrest, followed by inflammatory, infectious and traumatic conditions of the major organs, hemorrhage/hemorrhagic shock, and healing of bone defects/fractures. Actually, there was a wider array of models directed, including various types of experimental burns, spinal cord injuries, ischemia-reperfusion injuries, and intoxication/poisoning. This was undoubtedly in parallel with the daily practices of emergency physicians who treat a broad spectrum of illnesses and who should have knowledge touching many different disciplines.

On the other hand, despite the fact that almost one-third of the evidence obtained from experimental research in the field of EM was provided by emergency department physicians, the contribution of others such as anesthesiology, orthopedics, and general surgery was appreciable. This could naturally be explained by the high frequency of emergent cases encountered in these branches, leading the physicians to look for better solutions. Excitingly, preclinical sciences, which cover a great number of subfields, were the fourth most common contributors to experimental EM literature. This was indeed encouraging as an indicator of high interest in EM topics and also EM journals by other disciplines.

Rodents are small animals that can rapidly increase in number and live short lives. Consequently, easy handling and housing along with their relatively cheap costs besides commonalities with human genetics and biology render them the most preferred subjects of experimental studies [17]. Alternatively, swine are widely used in research as well, given their anatomical, biological, and physiological characteristics resembling those of humans [17, 18]. Consistent with this general tendency, the majority of the studies assessed in our report used rodents followed by swine, constituting together more than 85% of all subject animals.

Among all, cardiac arrest was the most common performed injury model while in a decreasing order of frequency; brain, hemodynamic parameters, and heart were the most examined targets in the points of how they were affected or how they responded. Since the injury models primarily implemented on the brain were not that popular, this can be interpreted as the ongoing concerns and challenges of physicians regarding the neurological consequences of certain pathophysiologic situations, particularly cardiac arrest, which acutely influence the human body.

As outcome measures, more than 50% of the researches focused on clinical outcomes and/or survival rates. Besides, a substantial proportion of the studies supported their hypothesis with histopathological methods while a small group included the results of blood/serum analysis and radiologic evaluations. Importantly, only a very limited number of studies were aimed at the affected signaling pathways of the cellular processes involved. This could be explained by the tendency of authors to unveil the results of their procedures rather than the molecular mechanisms behind the occasion. Likewise, it could be explained by the trend in submitting research reports focusing on pathophysiology to the journals that are more familiar to preclinical sciences such as physiology, pharmacology, and biochemistry. instead of those closer to clinical sciences including EM. Nevertheless, it should be noted that better understanding of the development, progression and courses of diseases highly depend on comprehending the fundamentals of interactions between different cellular systems both in physiologic and pathologic conditions. Therefore, researchers should be encouraged to routinize questioning the underlying molecular mechanisms of their area of interest while also improving their scientific reliability.

Among all, Resuscitation published the highest number of articles and received the majority of the citations, followed by Injury, Turkish Journal of Trauma and Emergency Surgery, and American Journal of Emergency Medicine. Today, the impact factor of the publishing journal is accepted as the primary indicator of future citations of an article [19, 20]. In other words, works published in higher impact journals are expected to be cited more. In our study, the total citation counts of the journals seemed to increase in line with their total number of articles as well. For example, Injury, Turkish Journal of Trauma and Emergency Surgery, and American Journal of Emergency Medicine received more citations than the journals with relatively higher impact factors, probably due to their high number of articles.

The USA had the largest contribution in consistence with former reports which in fact revealed Canada and Australia as following [3, 4]. Contrarily, according to our analysis, Turkey and China were the next leading countries in publishing experimental animal study reports after the USA. In addition, the USA received more than one-third of the citations followed by China and Turkey. To be noted, previous bibliometric studies used the citation counts of the most cited EM articles to rank the contributor countries but not their total number of published papers on a certain topic [3, 4]. These disparities could have also resulted from further factors such as the differences between countries in their prominent interest fields of EM, either clinical or experimental research, or their distinct journal preferences for submitting their clinical and experimental studies. Nonetheless, since our ultimate goal was not to compare the number of articles as well as the citation status of the journals and the countries, we did not perform statistical analysis in this regard, but basically interpreted the results of our appraisal.

Above all, the recent decrease in the number of articles was perhaps the most important finding. This could have emerged from the low affinity of EM journals for experimental reports or the low intention of researchers either to perform experimental studies or to publish them in the journals that counted under EM. Regardless, we believe EM journals should encourage and consider giving more place to experimental research given their undisputed worth and potential future contributions to science, including the field of EM.

Manually examining each issue of the participant journals meticulously was the strength of our study. This method likely precluded missing articles, which is a possibility in web-based searches using keywords. Moreover, analysis depending on highly cited articles tends to favor older articles, limits the scope of the search to an intended point such as 50, 100, etc., and has a potential to overlook the topics which did not gain enough citations to merit a place in the top cited lists. In this view, evaluating the journals and articles one by one possibly minimalized the bias associated with search methods while also allowing a summarized and condensed review of all titles that have been addressed so far.

Conversely, several unavoidable limitations due to the bibliometric basis of this work should be contemplated while considering the results. First, we did not perform an independent search to eliminate self-citations, which may fictitiously exaggerate the actual influence of the articles. Lack of statistical analysis can also be deliberated as a limitation. However, as mentioned earlier, it would be a challenge with the primary purpose of this study. Lastly, our analysis was limited to the articles published in SCI-E EM journals. Emergency medicine literature obviously extends beyond the borders of the abovementioned journals since authors do not always publish their studies in the journals of their own fields but sometimes elect to share their data through the journals of other territories. In this regard, a more comprehensive search including experimental animal research studies performed primarily by EM authors and subsequently published in journals other than EM journals could provide a wider opinion; however, such an analysis is beyond the scope of the current study as our primary aim was to focus on the experimental research published in EM journals to expose the propensities, distribution, and shortcoming topics.

## 5. Conclusions

Until today, bibliometric analysis regarding EM has mostly focused on the highly cited articles of the general literature, causing less cited but still crucial topics including experimental research to be unnoted. With the present study, we provided the featuring headlines and distribution of the experimental animal studies in the EM territory. Unfortunately, the majority of the field journals were found not to give enough space to these studies. We hope that future experimental studies respecting EM will be encouraged by the augmented number of reports published in prominent EM journals while supporting the inevitable contribution of animal research to the evolution of science.

## Figures and Tables

**Table 1:** Distribution of the number of articles and their citation status according to the journals.

Journal Name	Impact Factor	Number of Articles	Number of Citations	Citation Rank
Resuscitation	5.863	285	3787	1
Injury	2.199	143	1588	2
Turkish Journal of Trauma and Emergency Surgery	0.525	128	455	3
American Journal of Emergency Medicine	1.29	63	451	4
Academic Emergency Medicine	2.612	37	426	5
Annals of Emergency Medicine	4.680	11	179	6
Scandinavian Journal of Trauma, Resuscitation and Emergency Medicine	2.312	11	47	9
European Journal of Trauma and Emergency Surgery	1.704	10	9	15
Prehospital Emergency Care	2.269	9	67	8
Burns & Trauma	NA	8	19	12
Journal of Emergency Medicine	1.207	8	98	7
Hong Kong Journal of Emergency Medicine	0.202	5	14	13
Signa Vitae	1.26	5	5	16
World Journal of Emergency Surgery	3.198	4	20	11
BMC Emergency Medicine	2.046	3	24	10
European Journal of Emergency Medicine	1.729	3	14	14
Prehospital and Disaster Medicine	0.971	2	4	17
Emergencias	3.608	1	0	18

*NA:* Not available.

**Table 2:** Distribution of the articles and their citation status according to countries.

Country	Number of Articles (%)	Number of Citations (%)	Citation Rank
USA	199 (27)	2663 (37)	1
Turkey	147 (20)	757 (10.5)	3
China	102 (13.9)	1102 (15.3)	2
Germany	58 (7.9)	544 (7.5)	4
Korea	24 (3.3)	178 (2.5)	7
Taiwan	21 (2.9)	332 (4.6)	5
Austria	20 (2.7)	232 (3.2)	6
Spain	14 (1.9)	108 (1.5)	13
Greece	14 (1.9)	105 (1.5)	14
France	13 (1.8)	115 (1.6)	11
Brazil	13 (1.8)	106 (1.5)	12
Sweden	12 (1.6)	143 (2)	8
Canada	11 (1.5)	89 (1.2)	15
Iran	10 (1.4)	42 (0.6)	17
Japan	9 (1.2)	88 (1.2)	16
Norway	9 (1.2)	127 (1.8)	9
Switzerland	8 (1)	124 (1.7)	10
Others	52 (7)	352 (4.9)	-

**Table 3:** Characteristics of the experimental studies subjected in the publications.

	Variables	Number of studies (%)
Department of the First Author	Emergency Medicine	190 (28.8)
	Anesthesiology and Critical Care	107 (16.2)
	Orthopedics and Traumatology	72 (10.9)
	Surgery	64 (9.7)
	Preclinical Sciences	37 (5.6)
	Pediatrics	28 (4.2)
	Neurosurgery	26 (4)
	Burn, Plastic and Reconstructive Surgery	24 (3.7)
	Cardiovascular	21 (3.2)
	Biomechanical or Biomedical Engineering	13 (2)
	Cardiothoracic Surgery	7 (1)
	Experimental Surgery and Research	7 (1)
	Pathology	7 (1)
	Others	58 (8.8)

Subject Species	Rodent	367 (49.9)
	Swine	272 (37)
	Rabbit	52 (7.1)
	Sheep	20 (2.7)
	Dog	13 (1.8)
	Others	12 (1.5)

Experimental Model	Cardiac Arrest	257 (34.9)
	Inflammation/Infection/Ischemia/Trauma of Major Organs	87 (11.8)
	Hemorrhage/Hemorrhagic Shock/Coagulation	81 (11)
	Bone Defects/Fracture Healing	66 (9)
	Burn Injury	30 (4.1)
	Spinal Cord Injury	26 (3.5)
	Ischemia/Reperfusion	23 (3.1)
	Intoxication/Poisoning	23 (3.1)
	Peripheral Nerve Injury	22 (3)
	Therapeutic Hypothermia	17 (2.3)
	Wound Healing	16 (2.2)
	Sepsis	15 (2)
	Cerebral Ischemia	14 (1.9)
	Traumatic Brain Injury	13 (1.8)
	Technique-Training	12 (1.6)
	Muscle/Tendon/Ligament Injury	11 (1.5)
	Others	23 (3.1)

Target Organ/System Evaluated	Brain	101 (13.7)
	Hemodynamic Parameters	94 (12.8)
	Heart	80 (10.9)
	Bones	61 (8.3)
	Lungs	51 (6.9)
	Spinal Cord	26 (3.5)
	Peripheral Nerves	23 (3.1)
	Skin	21 (2.9)
	Coagulation System	20 (2.7)
	Kidneys	17 (2.3)
	Musculoskeletal System	17 (2.3)
	Intestines	9 (1.2)
	Liver	8 (1.1)
	Multiple Organs	23 (3.1)
	Others	185 (25.1)

Type of the Evaluation Method	Outcome/survival	408 (55.4)
	Histopathological	354 (48.1)
	Blood/serum	124 (16.8)
	Radiologic	36 (4.9)
	Polymerase Chain Reaction	25 (3.4)
	Electrophysiologic	12 (1.6)

**Table 4:** The most influential experimental animal studies in the emergency medicine literature.

Rank	Journal	Year of Publication	First Author	Title	Number of Citations	Country
1	Resuscitation	2008	Jia X.	Improving neurological outcomes post-cardiac arrest in a rat model: Immediate hypothermia and quantitative EEG monitoring	86	USA

2	Resuscitation	2008	Zhao D.	Intra-arrest cooling with delayed reperfusion yields higher survival than earlier normothermic resuscitation in a mouse model of cardiac arrest	78	USA

3	Injury	2010	Zhang X.	Chlorogenic acid protects mice against lipopolysaccharide-induced acute lung injury	76	China

4	Resuscitation	2009	Yang CH.	Dexmedetomidine–ketamine combination mitigates acute lung injury in haemorrhagic shock rats	59	Taiwan

5	Resuscitation	2010	White NJ.	Coagulopathy and traumatic shock: Characterizing hemostatic function during the critical period prior to fluid resuscitation	54	USA

6	Injury	2011	Adamskaya N.	Light therapy by blue LED improves wound healing in an excision model in rats	53	Austria

7	Injury	2011	Kanthan SR.	Platelet-rich plasma (PRP) enhances bone healing in non-united critical-sized defects: A preliminary study involving rabbit models	51	Malaysia

8	Injury	2009	Klaue K.	Bone regeneration in long-bone defects: tissue compartmentalization? In vivo study on bone defects in sheep	49	Switzerland

9	Resuscitation	2013	Sutton RM.	Hemodynamic directed CPR improves short-term survival from asphyxia-associated cardiac arrest	47	USA

10	Resuscitation	2008	Angelos Mark G.	Cardiovascular response to epinephrine varies with increasing duration of cardiac arrest	45	USA

11	Resuscitation	2010	Gotberg M.	Mild hypothermia reduces acute mortality and improves hemodynamic outcome in a cardiogenic shock pig model	45	Sweden

12	Resuscitation	2012	Hoskins SL.	Pharmacokinetics of intraosseous and central venous drug delivery during cardiopulmonary resuscitation	44	USA

13	Injury	2010	Hakimi M.	Combined use of platelet-rich plasma and autologous bone grafts in the treatment of long bone defects in mini-pigs	44	Germany

14	Acad Emerg Med	2008	Perez E.	Determining the Optimal Dose of Intravenous Fat Emulsion for the Treatment of Severe Verapamil Toxicity in a Rodent Model	43	USA

15	Am J Emerg Med	2009	Wu JY.	A comparison of 2 types of chest compressions in a porcine model of cardiac arrest	42	China

16	Resuscitation	2012	Aksu U.	Balanced vs unbalanced crystalloid resuscitation in a near-fatal model of hemorrhagic shock and the effects on renal oxygenation, oxidative stress, and inflammation	42	Netherlands

17	Resuscitation	2010	Solevag AL.	Extended series of cardiac compressions during CPR in a swine model of perinatal asphyxia	42	Norway

18	Acad Emerg Med	2011	Littlejohn LF.	Comparison of Celox-A, ChitoFlex, WoundStat, and Combat Gauze Hemostatic Agents Versus Standard Gauze Dressing in Control of Hemorrhage in a Swine Model of Penetrating Trauma	41	USA

19	Ann Emerg Med	2010	Niiya T.	Intravenous Lipid Emulsion Sequesters Amiodarone in Plasma and Eliminates Its Hypotensive Action in Pigs	40	Finland

20	Resuscitation	2010	Wang H.	Intra-arrest selective brain cooling improves success of resuscitation in a porcine model of prolonged cardiac arrest	39	USA

21	Resuscitation	2008	Ristagno G.	Cerebral cortical microvascular flow during and following cardiopulmonary resuscitation after short duration of cardiac arrest	39	USA

22	Resuscitation	2008	Hutchens MP.	Soluble epoxide hydrolase gene deletion reduces survival after cardiac arrest and cardiopulmonary resuscitation	39	USA

23	Resuscitation	2014	Osredkar D.	Hypothermia is not neuroprotective after infection-sensitized neonatal hypoxic-ischemic brain injury	38	Norway

24	Resuscitation	2012	Kim K.	Effect of valproic acid on acute lung injury in a rodent model of intestinal ischemia reperfusion	37	USA

25	Ann Emerg Med	2010	Bebarta VS.	Hydroxocobalamin and Sodium Thiosulfate Versus Sodium Nitrite and Sodium Thiosulfate in the Treatment of Acute Cyanide Toxicity in a Swine (Sus scrofa) Model	37	USA

## Data Availability

Data will be provided on request through the corresponding author of this article.
